# Exhaustion of mitochondrial and autophagic reserve may contribute to the development of *LRRK2*^*G2019S*^-Parkinson’s disease

**DOI:** 10.1186/s12967-018-1526-3

**Published:** 2018-06-08

**Authors:** Diana Luz Juárez-Flores, Ingrid González-Casacuberta, Mario Ezquerra, María Bañó, Francesc Carmona-Pontaque, Marc Catalán-García, Mariona Guitart-Mampel, Juan José Rivero, Ester Tobias, Jose Cesar Milisenda, Eduard Tolosa, Maria Jose Marti, Ruben Fernández-Santiago, Francesc Cardellach, Constanza Morén, Glòria Garrabou

**Affiliations:** 1 Laboratory of Muscle Research and Mitochondrial Function-CELLEX, Institut d‘Investigacions Biomèdiques August Pi i Sunyer (IDIBAPS), Department of Internal Medicine-Hospital Clínic of Barcelona, Faculty of Medicine and Health Sciences, University of Barcelona (UB), Barcelona, Spain; 2 Centro de Investigación Biomédica en Red de Enfermedades Raras (CIBERER), Madrid, Spain; 3 Laboratory of Parkinson disease and other Neurodegenerative Movement Disorders: Clinical and Experimental Research, Department of Neurology, Hospital Clínic of Barcelona, Institut d’Investigacions Biomèdiques August Pi i Sunyer (IDIBAPS), University of Barcelona (UB), Barcelona, Spain; 4 CIBER de Enfermedades Neurodegenerativas (CIBERNED), Madrid, Spain; 5 Department of Genetics, Microbiology and Statistics, University of Barcelona, Barcelona, Spain

**Keywords:** Parkinson’s disease, *LRRK2*, *G2019S*, Non-manifesting carriers, Mitochondrial dysfunction, Mitochondrial dynamics, Autophagy, Fibroblasts, Glucose, Galactose

## Abstract

**Background:**

Mutations in *leucine rich repeat kinase 2* (*LRRK2*) are the most common cause of familial Parkinson’s disease (PD). Mitochondrial and autophagic dysfunction has been described as etiologic factors in different experimental models of PD. We aimed to study the role of mitochondria and autophagy in *LRRK2*^*G2019S*^-mutation, and its relationship with the presence of PD-symptoms.

**Methods:**

Fibroblasts from six non-manifesting *LRRK2*^*G2019S*^-carriers (NM-*LRRK2*^*G2019S*^) and seven patients with *LRRK2*^*G2019S*^-associated PD (PD-*LRRK2*^*G2019S*^) were compared to eight healthy controls (C). An exhaustive assessment of mitochondrial performance and autophagy was performed after 24-h exposure to standard (glucose) or mitochondrial-challenging environment (galactose), where mitochondrial and autophagy impairment may be heightened.

**Results:**

A similar mitochondrial phenotype of NM-*LRRK2*^*G2019S*^ and controls, except for an early mitochondrial depolarization (54.14% increased, *p *= 0.04), was shown in glucose. In response to galactose, mitochondrial dynamics of NM-*LRRK2*^*G2019S*^ improved (− 17.54% circularity, *p *= 0.002 and + 42.53% form factor, *p *= 0.051), probably to maintain ATP levels over controls. A compromised bioenergetic function was suggested in PD-*LRRK2*^*G2019S*^ when compared to controls in glucose media. An inefficient response to galactose and worsened mitochondrial dynamics (− 37.7% mitochondrial elongation, *p *= 0.053) was shown, leading to increased oxidative stress. Autophagy initiation (SQTSM/P62) was upregulated in NM-*LRRK2*^*G2019S*^ when compared to controls (glucose + 118.4%, *p *= 0.014; galactose + 114.44%, *p *= 0.009,) and autophagosome formation increased in glucose media. Despite of elevated SQSTM1/P62 levels of PD-*NM*^*G2019S*^ when compared to controls (glucose + 226.14%, p = 0.04; galactose + 78.5%, p = 0.02), autophagosome formation was deficient in PD-*LRRK2*^*G2019S*^ when compared to NM-*LRRK2*^*G2019S*^ (− 71.26%, p = 0.022).

**Conclusions:**

Enhanced mitochondrial performance of NM-*LRRK2*^*G2019S*^ in mitochondrial-challenging conditions and upregulation of autophagy suggests that an exhaustion of mitochondrial bioenergetic and autophagic reserve, may contribute to the development of PD in *LRRK2*^*G2019S*^ mutation carriers.

**Electronic supplementary material:**

The online version of this article (10.1186/s12967-018-1526-3) contains supplementary material, which is available to authorized users.

## Background

Parkinson’s disease (PD) is the second most prevalent neurodegenerative disease. While most of PD cases are idiopathic, monogenic forms of the disease are demonstrated in about 5–10% of patients [1]. Mutations in *leucine*-*rich repeat kinase 2* (*LRRK2)* are the most common cause of inherited PD and account for 1–2% of sporadic PD cases [2]. LRRK2 is a large multi-domain protein with kinase and GTPase activity involved in several cellular functions [3]. A glycine to serine substitution in the kinase domain of this protein (*G2019S*), accounts for the majority of genetically-transmitted, late-onset PD and for a variable proportion of sporadic PD, with higher prevalence in Ashkenazi Jews and North-African populations [4, 5]. Previous experimental models of PD have reported that LRRK2 mutations play a role in α-synuclein phosphorylation and depot, microtubule dynamics regulation, alterations in the ubiquitin–proteasome system and in neurite growth and branching of neurons [6].

Current evidence suggests that the increased kinase activity caused by the *LRRK2*^*G2019S*^-mutation (*LRRK2*^*G2019S*^), associated with the regulation of mitochondrial dynamics, vesicle trafficking and chaperone mediated autophagy [7–9], plays a pivotal role in the pathogenesis of *LRRK2*^*G2019S*^-associated PD, but the molecular mechanisms leading to neurodegeneration remain largely unknown [10].

Neuronal function is strongly dependent on oxidative metabolism and efficient organelle clearance, and mitochondrial homeostasis greatly depends on adequate mitochondrial dynamics, turnover and renewal through autophagy, which makes the study of mitochondrial function and autophagy highly relevant in the elucidation of pathological pathways leading to neurodegeneration [7, 8]. Recent studies have characterized mitochondrial dysfunction and autophagy impairment in *LRRK2*^*G2019S*^-associated PD [9–11]. Furthermore, therapeutic strategies in *LRRK2*^*G2019S*^-associated PD consisting of drug testing in experimental models directed to reverse mitochondrial alterations, and LRRK2 inhibition as a therapeutic target for impaired autophagy in PD, have been tested with positive outcomes [12–14]. However, whether mitochondrial and autophagic alterations are cause or effect of the pathophysiology of *LRRK2*^*G2019S*^ PD is still unclear.

In this regard, the study of *LRRK2*^*G2019S*^-carriers without PD symptoms (NM-*LRRK2*^*G2019S*^) in contrast to *LRRK2*^*G2019S*^-carriers diagnosed with PD (PD-L*RRK2*^*G2019S*^), provides the opportunity to investigate early molecular alterations in this condition [6]. Motor symptoms of PD appear when loss of more than 60–70% of dopaminergic neurons has occurred, preceded by several years of neurodegeneration at different levels, and followed by a rapid progression of the disease [15]. In the last decade, research has focused on clinical and molecular alterations in the prodromal phase of PD [16] and in asymptomatic carriers of PD-linked mutations [14, 17], all of which has raised a great interest in the search of the disease aetiology, novel biomarkers and potential targets to prevent and modify the natural history of the disease.

Since the central nervous system is not readily available for investigation, several experimental models have been developed in the pursuit of unravelling PD pathophysiology [15, 16]. Past studies have provided evidence of molecular alterations of PD in peripheral tissues [18–20], such as skin-derived fibroblasts, where defined mutations and cumulative cellular damage of donors are present [21]. Concerns have risen about the use of toxic insults and non-physiologic metabolic conditions in previous cell or animal models of the disease, which may not reproduce the mitochondrial and autophagic derangements that lead to neurodegeneration. Glucose promotes anaerobic glycolysis in detriment of oxidative mitochondrial metabolism, thus masking potential bioenergetic alterations and consequent autophagic fails which may be more evident in mitochondrial-challenging conditions. In this sense, the use of galactose has been proved useful in the study of primary mitochondrial diseases, since it enhances mitochondrial metabolism, evidencing pre-existent mitochondrial alterations [22] and autophagic derangements.

In the present study, we hypothesized that mitochondrial and autophagic alterations represent a primary and systemic process in PD, which may play a role in the development of symptoms of *LRRK2*^*G2019S*^-associated PD, and that such derangements may be exacerbated in mitochondrial-challenging conditions. Consequently, we evaluated the mitochondrial and autophagic phenotype in fibroblasts from NM-*LRRK2*^*G2019S*^ subjects and PD-L*RRK2*^*G2019S*^ patients, either in glycolytic or oxidative conditions enabled by the use of galactose.

## Methods

### Study design and population

A single-site, cross-sectional, observational study was conducted. Twenty-one age and gender paired subjects were included: six NM-*LRRK2*^*G2019S*^, seven PD-*LRRK2*^*G2019S*^, and eight healthy, unrelated controls (C). All PD-*LRRK2*^*G2019S*^ patients met the UK Brain Bank Criteria for PD [23]. Control group included *LRRK2*^*G2019S*^ negative relatives of patients who voluntarily underwent skin biopsy. Subjects with comorbidities, mitochondrial disorders, and those consuming mitochondrial toxic drugs were not included in any of those groups [24].

### Fibroblasts culture

Fibroblasts were obtained by a skin punch biopsy and mutation screening was performed as previously described [5].

Cells were grown in 25 mM glucose DMEM medium (Gibco, Life Technologies) supplemented with 10% heat-inactivated fetal bovine serum and 1% penicillin–streptomycin at 37 °C, in a humidified 5% CO_2_ air incubator, until 80% optimal confluence was reached. In order to assess whether mitochondrial and autophagic function was directly implicated in the pathogeny of *LRRK2*^*G2019S*^, cells were exposed for 24-h to either 25 mM glucose (standard) or 10 mM galactose (mitochondrial-challenging) media [22], where cells are forced to rely on oxidative phosphorylation for ATP production. Fibroblasts were harvested with 2.5% trypsin, (Gibco, Life technologies™) at 500 g for 8 min. In vivo experiments, including oxygen consumption and mitochondrial membrane potential (MMP), were performed in parallel including one subject from each cohort, at the same passage, both in glucose and galactose media. Fixation of cells for immunofluorescent quantification of mitochondrial dynamics was also performed at this time point. Cell pellets from each line were kept at − 80 °C for further experimental procedures. All functional assays were performed in cells between passage 5 and 10.

### Experimental parameters

In order to address an exhaustive mitochondrial phenotyping and autophagic print, this study contemplated the following experiments:

#### Mitochondrial phenotype

- *Mitochondrial DNA, RNA and protein* content:
  Total DNA was extracted by standard phenol chloroform procedure and total RNA was extracted by affinity microcolumns and retro transcribed to cDNA in triplicates, as reported elsewhere [25]. Mitochondrial DNA and RNA content were measured as the ratio between a mtDNA encoded gene/transcript and a nuclear encoded one (mt12SrRNA/nRNAseP). Mitochondrial protein content was assessed by Western Blot in duplicates, through 7/13% SDS-PAGE and immunoquantification of nuclear-encoded VDAC, mitochondrial-encoded COXII, and nuclear-encoded COXIV analysis, normalized by β-actin. Chemiluminescence was quantified with ImageQuantLD® [26].
- *Mitochondrial function:*
  i. Mitochondrial respiratory chain (MRC) enzyme activities previously associated to PD [14] were measured by spectrophotometry, following Spanish standardized national procedures (unpublished data). Complex I (CI) and Complex IV (CIV) enzyme activities were then normalized by citrate synthase (CS), widely considered as a reliable marker of mitochondrial content.
  ii. Mitochondrial respiration is the result of oxygen consumption by the MRC. CI stimulated oxygen consumption was assessed through pyruvate-malate oxidation (PMox) by high-resolution respirometry using Oroboros™ Oxygraph-2K® (Innsbruck, Austria) in permeabilized fibroblasts, following manufacturer protocols [27].
  iii. Total cellular ATP was measured in duplicates by using the Luminescent ATP detection assay kit® #ab113849; Abcam™ (Cambridge, United Kingdom), according to manufacturer’s instructions, in order to quantify the bioenergetic efficiency of MRC.
  iv. Mitochondrial damage markers were measured to search for mitochondrial lesion in association with the presence of *LRRK2*^*G2019S*^ Mitochondrial membrane potential was measured by flow cytometry twice to establish whether punctual mitochondrial alterations in MMP prelude mitochondrial dysfunction, as previously described [28]. Second, lipid peroxidation was measured in duplicates by the spectrophotometric measurement of malondialdehyde (MDA) and 4-hydroxyalkenal (HAE), as indicators of oxidative damage of reactive oxygen species (ROS) into cellular lipid compounds, as reported elsewhere [29]. Finally, to evaluate the apoptotic rate, cells were double-stained for annexin V and propidium iodide and quantified by flow cytometry, as previously reported [30].
- *Mitochondrial dynamics* has been shown to be crucial in the maintenance of mitochondrial homeostasis, and LRRK2 has been suggested to play a role in mitochondrial fission [31]. Immunocytochemistry using confocal microscopy was performed as previously described [32]. One cell from three different fields for each cell line were randomly selected and analyzed with Image J [33] software to quantify the following parameters of mitochondrial dynamics:
  i. Mitochondrial network or mitochondrial content: Total number of mitochondria/total cell area [34]; higher mitochondrial network values are considered a sign of healthy mitochondria.
  ii. Circularity (Circ) or mitochondrial isolation: 4π.area/perimeter^2^; circular mitochondria have less interaction sites with other mitochondria, thus, Circ = 1 refers to poor mitochondrial dynamics of isolated mitochondria.
  iii. Aspect ratio (AR) or mitochondrial elongation: major/minor axis, AR = 1 indicates a perfect circle; AR increases as mitochondria elongate and become more elliptical, which is considered a beneficial sign of mitochondrial dynamics.
  iv. Form factor (FF) or mitochondrial branching: Circ-1; FF = 1 corresponds to a circular, unbranched mitochondrion and high FF values indicate branched and connected mitochondria, which is favourable for mitochondrial function.

#### Autophagic print

Characterization of autophagic print was carried out twice by quantitative measurement of autophagic-related proteins in both media (basal). To further assess autophagosome formation, 100 nM Bafilomycin A1 was added at two different time points (4 and 8 h) [35]. Briefly, fibroblasts were lysed with RIPA buffer and protease inhibitors followed by centrifugation at 14,000*g* at 4 °C for 5 min. Soluble fraction was retained for Western Blot analysis. Equal protein load lysates were resolved through 7/15% SDS-PAGE and transferred into nitrocellulose membranes, following blocking with 10% skimmed milk. Membranes were hybridized with Anti-SQSTM1/p62 and anti-LC3B antibodies overnight at 4 °C. Protein expression was normalized by β-actin protein content in all cases, as a cell loading control. Chemiluminescence was quantified with ImageQuantLD® and results were expressed and interpreted as:

i. Autophagy substrate; (SQSTM/p62)/β-actin is a cargo protein widely used as a marker of autophagy initiation [13].
ii. Autophagy receptor (LC3BI/β-actin) and lipidated form of the autophagy receptor (LC3BII/β-actin): LC3B is a marker of autophagosomes; LC3BI is unspecific and expressed in the autophagosome membrane but can also be located in cytoplasm or other organelles, its lipidation and migration to the autophagosome membrane (LC3BII) is considered to be a marker of autophagosome formation [36].

Immunocytochemistry was performed to further characterize autophagosome formation in the basal state, in glucose and galactose media, and after 6 h exposure to 100 nM Bafilomycin A1 using confocal microscopy. Briefly, cultured skin fibroblasts were washed with PBS before fixation with 4% paraformaldehyde for 15 min. Fixed cells were washed, permeabilized with 0.1% Digitonin in blocking solution (1% bovine serum albumin). Cells were incubated for 1 h using Anti-LC3 pAB. Counterstaining with DAPI was performed for nucleus visualization.

Experimental parameters were normalized by protein levels, measured by the BCA assay in quadruplicates, when needed. See Additional file 1: Material and methods for detailed protocols and reagents.

### Statistical analysis

Results were expressed as mean ± SEM and as a percentage of increase/decrease with respect to controls, which were arbitrarily assigned as a 0% baseline. Two statistical approaches were performed for each media (glucose or galactose): (i) Non-parametric Mann–Whitney analysis for independent samples to detect inter-group differences for individual parameters and (ii) principal component analysis (PCA) to define the component with the largest possible variance and to show whether the studied population would be clustered by group, age or sex. Statistical analysis was performed with SPSS 20.00 (for Mann–Whitney test) and the R programming language version 3.4.3 and its missMDA, package (for PCA) [37]. Statistical significance was set at *p *< 0.05 in all cases.

## Results

Epidemiological data of the studied cohorts at the time of skin biopsy are shown in Table 1 and clinical characteristics of PD-*LRRK2*^*G2019S*^ patients are shown in Additional file 2: Table S1. As expected, no significant differences in age and gender between groups were evidenced by either Mann–Whitney or PCA tests. None of the NM-*LRRK2*^*G2019S*^ subjects had developed clinical symptoms of the disease at the time of results analysis. Groups were not differentially clustered nor variables correlated among each other by performing PCA (Additional file 3: Figure S1).

**Table 1:** Epidemiological characteristics of the cohorts

Group	N	Gender		Age (years)		
		Male	Female	Range	Mean	SEM
NM-*LRRK2*^*G2019S*^	6	3 (50%)	3 (50%)	34–61	46.67	4.04
PD-*LRRK2*^*G2019S*^	7	3 (42.85%)	4 (57.15%)	44–71	60.57	3.15
Control	8	3 (37.50%)	5 (62.50%)	41–69	56.00	3.63

Epidemiological data of the studied cohorts at the time of skin biopsy. No significant differences were found in age or gender between groups

NM-*LRRK2*^*G2019S*^: Non-manifesting *LRRK2*^*G2019S*^-mutation carriers; PD-*LRRK2*^*G2019S*^: patients with *LRRK2*^*G2019S*^-mutation and clinically manifest PD; N: Number of cases enrolled; SEM: Standard error of the mean

### Mitochondrial phenotype

**Fig. 1:**
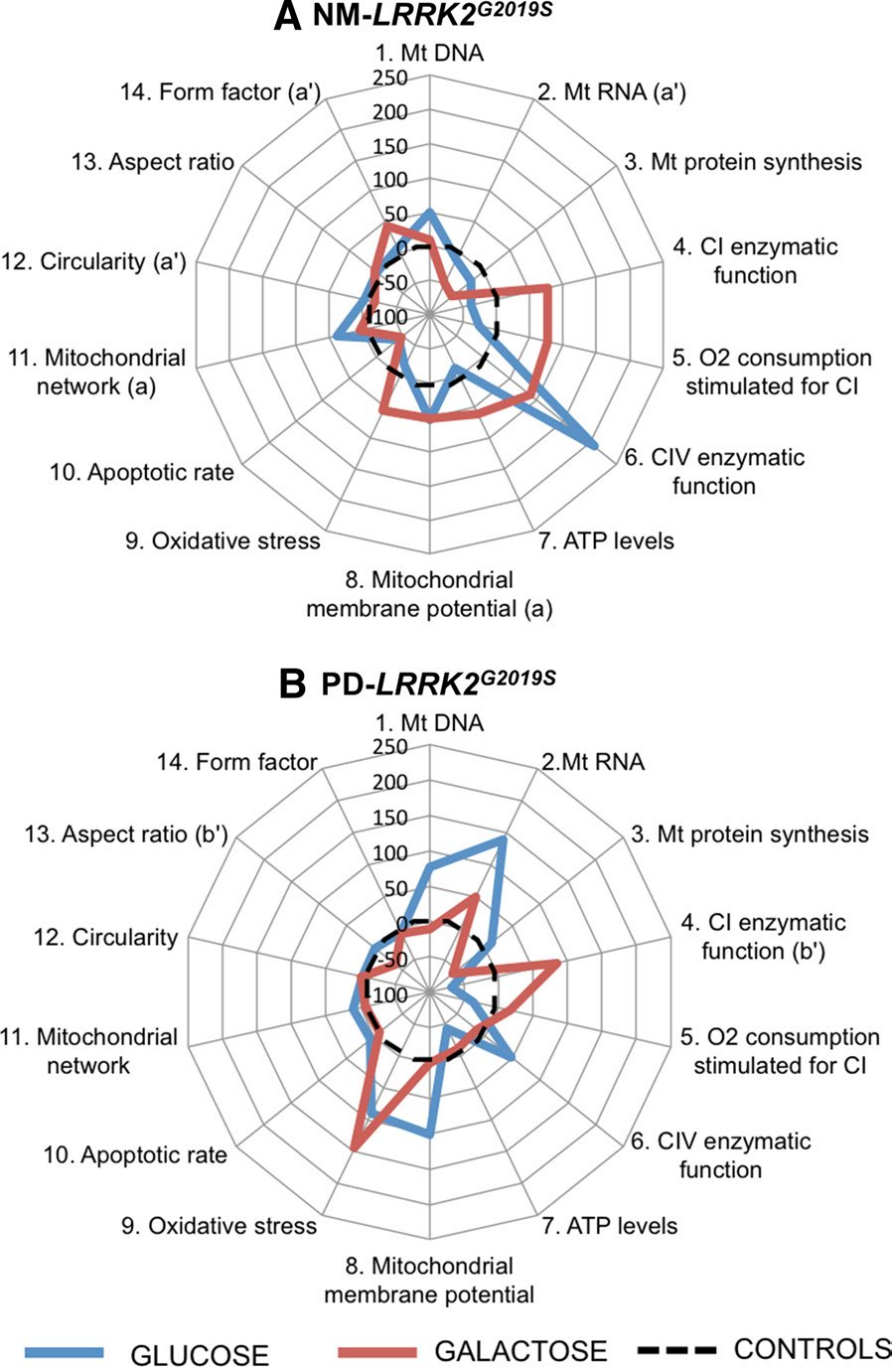
Mitochondrial phenotype of *LRRK2*^*G2019S*^-mutation carriers. Mitochondrial parameters measured in *LRRK2*^*G2019S*^-mutation carriers, without clinical symptoms of PD (NM-*LRRK2*^*G2019S*^) or with clinically manifest PD (PD-*LRRK2*^*G2019S*^) represented as a percentage of decrease/increase when compared to healthy controls, arbitrarily assigned as 0% (black, dotted line) in glucose (blue line) and galactose media (red line). The letters (a), (a’), (b) and (b’), indicate those cases in which a statistical difference within the two analysed groups and controls was found: (a) p < 0.05 when comparing NM-*LRRK2*^*G2019S*^ to controls in glucose media, (a’) p < 0.05 when comparing NM-*LRRK2*^*G2019S*^ to controls in galactose media, (b) p < 0.05 when comparing PD-*LRRK2*^*G2019S*^ to controls in glucose media and (b’) p < 0.05 when comparing PD-*LRRK2*^*G2019S*^ to controls in galactose media. In summary, fibroblasts of NM-*LRRK2*^*G2019S*^ showed a pattern similar to controls in glucose, except for early disruption of MMP. When subjected to mitochondrial challenging conditions, global mitochondrial function and dynamics trended to ameliorate, towards ATP production (**A**). Fibroblasts of PD-*LRRK2*^*G2019S*^ in glucose trended towards a deranged mitochondrial function when compared to controls. When subjected to galactose media, PD-*LRRK2*^*G2019S*^ fibroblasts presented an inefficient increase of mitochondrial function and worsened mitochondrial dynamics, leading to increased oxidative stress (**B**)

Figure 1 summarizes mitochondrial phenotype and raw data of experimental parameters is provided in Additional file 4: Table S2. Extended versions of graphics corresponding to genetics and mitochondrial protein synthesis can be found in Additional file 5: Figure S2.

- Mitochondrial function of NM-*LRRK2*^*G2019S*^ ameliorated when subjected to mitochondrial challenging conditions, in order to maintain ATP production over controls.
  Mitochondrial parameters were preserved in NM-*LRRK2*^*G2019S*^ subjects in glucose media with respect to controls except for early MMP depolarization (54.14%, p = 0.04, Figs. 1a.8 and 2f) and increased mitochondrial network (38.32%, p = 0.04, Figs. 1a.11 and 3b).

**Fig. 2:**
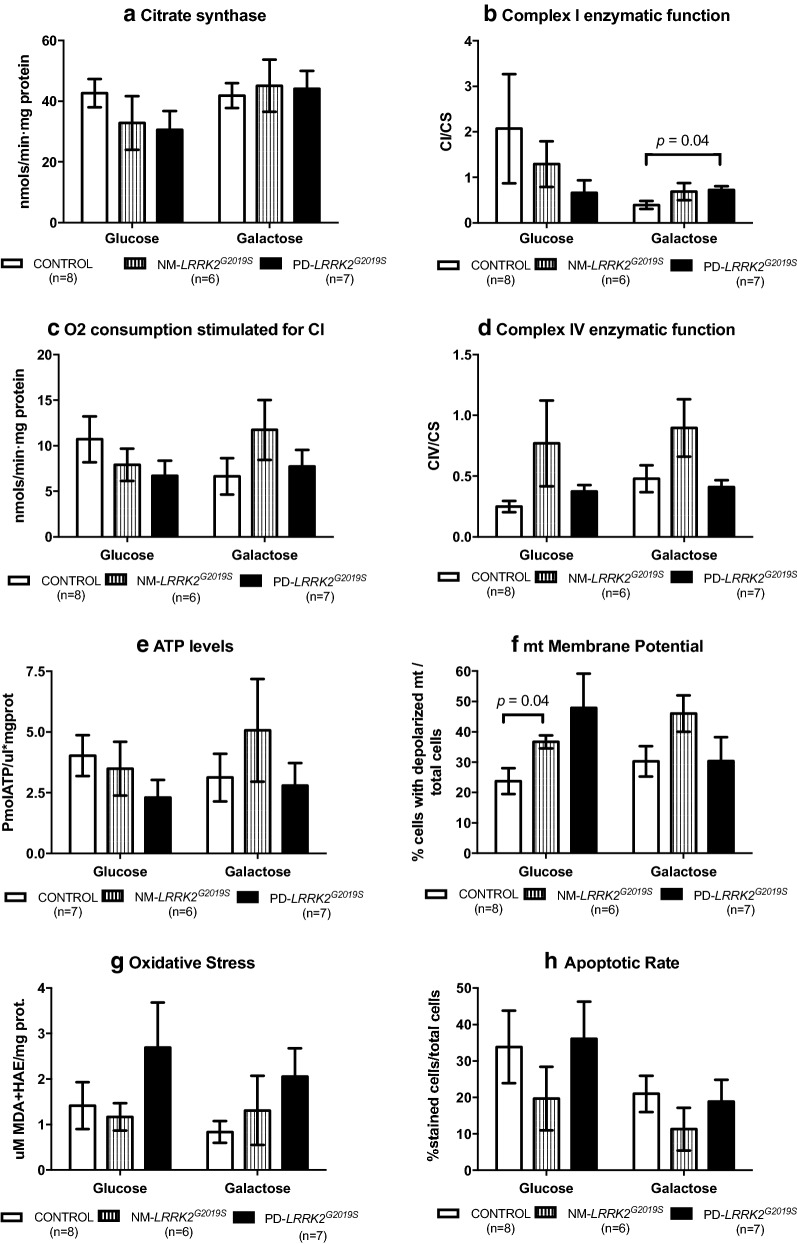
Mitochondrial function. Results are represented by mean ± SEM, comparing controls (n = 8; white bars), NM-*LRRK2*^*G2019S*^-mutation, (n = 6; grey bars) and PD-*LRRK2*^*G2019S*^ (n = 7; black bars) in glucose and galactose media. **a** Citrate synthase levels remained constant between groups in both media. **b** A trend to decrease CI enzymatic activity in NM-*LRRK2*^*G2019S*^ and PD-*LRRK2*^*G2019S*^ was observed in glucose media, and PD-*LRRK2*^*G2019S*^ patients harbouring the mutation increased their activity with respect to controls in galactose media (*p *= 0.04). **c** Oxygen consumption trended to reproduce the pattern of CI enzymatic activity. After exposure to galactose media, a non-significant increment in O_2_ consumption was observed in fibroblasts of NM-*LRRK2*^*G2019S*^ subjects with respect to controls (p = 0.35). **d** Complex IV enzymatic function remained similar in all groups, showing a trend to increase in NM-*LRRK2*^*G2019S*^ subjects with respect to controls in glucose and galactose media (*p *= 0.14 in both cases). **e** ATP levels tended to decrease in both groups in glucose media (p = 0.18 when comparing PD-*LRRK2*^*G2019S*^ with controls). **f** Number of depolarized mitochondria was increased in NM-*LRRK2*^*G2019S*^ when compared to controls in glucose media (p = 0.04), with the same trend observed for PD-*LRRK2*^*G2019S*^ although without reaching statistical significance (p = 0.14). **g** Oxidative damage trended to increase in PD-*LRRK2*^*G2019S*^ patients in both conditions when comparing them to controls. **h** Apoptotic rate tended to decrease in the NM-*LRRK2*^*G2019S*^ group in both media

**Fig. 3:**
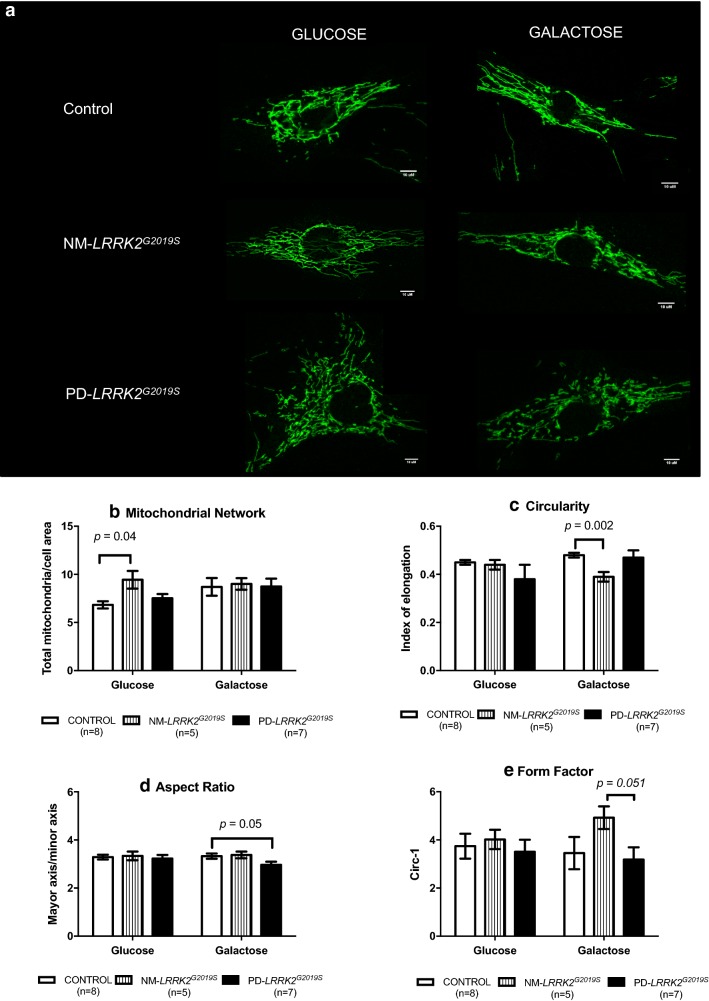
Mitochondrial dynamics. **a** Representative images of mitochondrial network obtained by confocal microscopy. NM-*LRRK2*^*G2019S*^: Non-manifesting carriers of *LRRK2*^*G2019S*^-mutation; PD-*LRRK2*^*G2019S*^: patients with *LRRK2*^*G2019S*^-mutation and clinically manifest PD. **b**–**e**. Results are represented by mean ± SEM, comparing controls (n = 8; white bars), NM-*LRRK2*^*G2019S*^ (n = 5, gray bars) and PD-*LRRK2*^*G2019S*^ (n = 7; black bars) in glucose and galactose media. Briefly, in fibroblasts of NM-*LRRK2*^*G2019S*^ a significant increase in mitochondrial network in standard conditions (glucose) was observed when compared to controls (**b**). When NM-*LRRK2*^*G2019S*^ fibroblasts were subjected to mitochondrial challenging conditions (galactose), an improvement of mitochondrial dynamics was seen, by decreased circularity (**c**) and trends to increase form factor (**e**), accounting for longer, more branched mitochondria. Fibroblasts of PD-*LRRK2*^*G2019S*^ showed a pattern similar to controls in standard (glucose) conditions and a handicapped response when exposed to mitochondrial challenging conditions (galactose) as shown by decreased aspect ratio (**d**), accounting for shorter mitochondria
  After galactose exposure, CI enzymatic function, O_2_ consumption stimulated for CI, CIV enzymatic function and ATP levels of NM-*LRRK2*^*G2019S*^ trended to overcome the level of controls (76.92, 76.87, 88.48 and 61.98%; *p *= 0.18, *p *= 0.35, *p *= 0.14 and *p *= 0.76, respectively), suggesting an adaptation to mitochondrial challenging conditions (Figs. 1a.4–7 and 2b–e). Mitochondrial dynamics ameliorated in fibroblasts of NM-*LRRK2*^*G2019S*^ with respect to controls when exposed to galactose medium, evidenced by 17.54% decreased circularity (*p *= 0.002) and 42.53% increased form factor (*p *= 0.051) (Fig. 1a.12, 14), accounting for longer, more branched mitochondria (Fig. 3c and e).
- PD-*LRRK2*^*G2019S*^ showed mitochondrial alterations in glucose media, which were worsened when subjected to mitochondrial-challenging conditions, resulting in increased oxidative stress.
  In fibroblasts from PD-*LRRK2*^*G2019S*^ patients in glucose media, CI enzyme function, O_2_ consumption stimulated for CI and ATP levels seemed to decrease with respect to controls (Figs. 1b.4, 5, 7 and 2b, c and e). Noticeably, although not significantly, depolarized mitochondria augmented to 101.34% (*p *= 0.14), accompanied by a 90% increase of oxidative stress (*p *= 0.46) with respect to controls (Figs. 1b.8, 9 and 2f, g).
  Exposure of PD-*LRRK2*^*G2019S*^ fibroblasts to galactose media increased CI enzyme function compared to controls (84.62%, *p *= 0.04), but such increase was neither translated into heightened oxygen consumption, nor enhanced CIV enzyme function or ATP synthesis (Figs. 1b.4, 7 and 2b–e), as formerly observed in asymptomatic carriers. Instead, oxidative damage increased 144.64% (*p *= 0.13) (Figs. 1b.9 and 2g) and, in detriment to mitochondrial dynamics, aspect ratio decreased 37.7% (*p *= 0.053) after exposure to mitochondrial challenging conditions (Figs. 1b.13 and 3d).

### Autophagy

**Fig. 4:**
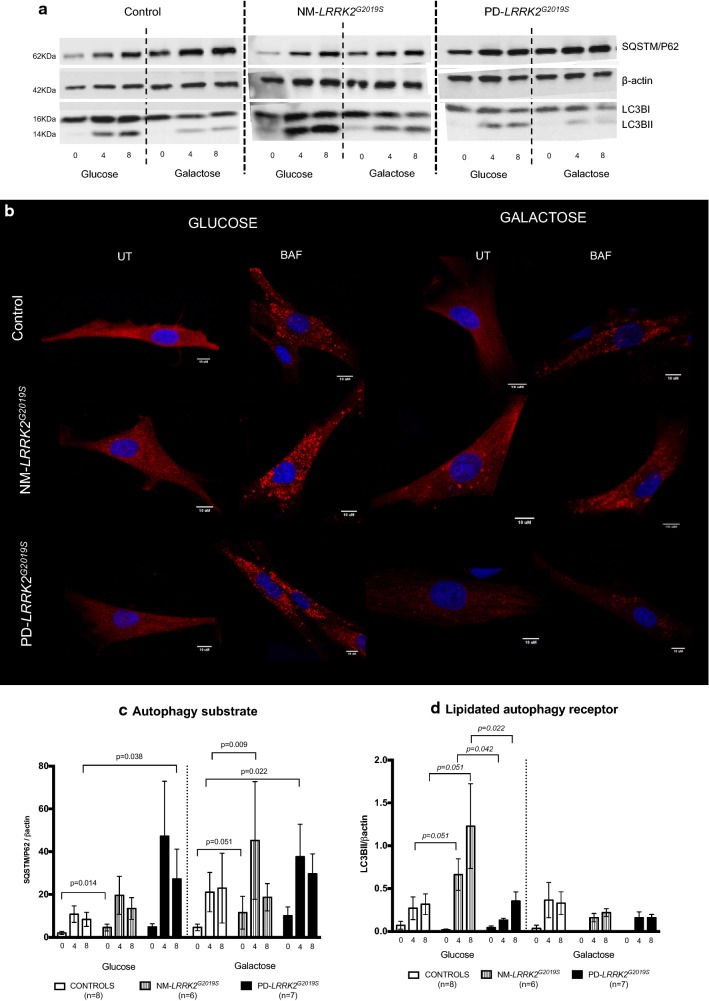
Autophagy. **a** Representative images (original images have been cropped and are available at request) of autophagy markers measured by Western blot comparing: NM-*LRRK2*^*G2019S*^: Non-manifesting carriers of *LRRK2*^*G2019S*^-mutation; PD-*LRRK2*^*G2019S*^: patients with *LRRK2*^*G2019S*^-mutation and clinically manifest PD. (SQSTM1/p62)/β-actin: Autophagy substrate, LC3B-I/β-actin: autophagy receptor, basal form. LC3B-II/β-actin: autophagy receptor, lipidated form. **b** Representative images of autophagosome formation (UT) and accumulation after 8 h of treatment with 100 nM bafilomycin (BAF) obtained by confocal microscopy. **c**, **d** Results are represented by mean ± SEM, comparing controls (n = 8; white bars), NM-*LRRK2*^*G2019S*^-mutation (n = 6, gray bars) and PD-*LRRK2*^*G2019S*^ (n = 7; black bars) in either glucose or galactose media and at basal state, and after 4 and 8 h treatment with bafilomycin (0, 4, 8). Autophagy initiation was upregulated in NM-*LRRK2*^*G2019S*^ subjects in glucose and galactose media when compared to controls (+ 118.4% SQSTM1/P62, p = 0.014 and + 114.44% SQSTM1/P62, p = 0.009, respectively) and trends to increase autophagy substrate were observed in PD-*LRRK2*^*G2019S*^ in both media (**c**). Autophagosome formation trended to increase at 4 and 8 h of bafilomycin treatment in NM-*LRRK2*^*G2019S*^ and was significantly decreased in PD-*LRRK2*^*G2019S*^ when compared to NM-*LRRK2*^*G2019S*^ subjects in glucose media at 4 and 8 h of bafilomycin treatment (− 79 to 86%, *p *= *0.042* and − 71.26%, *p *= *0.22* respectively) (**d**)

Figure 4 summarizes the autophagic characterization, and raw data of experimental parameters is provided in Additional file 4: Table S2.

- Autophagy initiation signalling increased in NM-*LRRK2*^*G2019S*^ subjects in glucose and galactose media, but autophagosome formation was only upregulated in glucose media.
  A significant increase in autophagy substrate expression (SQSTM1/p62) in fibroblasts of NM-*LRRK2*^*G2019S*^ was observed when compared to controls in glucose media (118.4%, *p *= *0.014*), and in galactose, after 4 h of bafilomycin exposure (114.44% increase, *p *= *0.009*) (Fig. 4c). These increased signalling for autophagy initiation was accompanied by trends to increase autophagosome formation at 4 and 8 h of bafilomycin treatment (LC3BII/βactin: + 145.22% and + 287.21%, respectively, *p *= *0.051*) when compared to controls in glucose media, but not in galactose media, where autophagosome formation seemed to be decreased (Fig. 4d).
- Autophagosome formation was decreased in PD-*LRRK2*^*G2019S*^ despite increased autophagy initiation in both media.
  Autophagy substrate expression was increased in PD-*LRRK2*^*G2019S*^ after 8 h of bafilomycin treatment in glucose media (226.14%., p = 0.04) and at 4 h in galactose media when compared to controls (78.5%, p = 0.02) (Fig. 4c). However, these increased signalling for autophagy initiation was not accompanied by autophagosome formation in any media, and a significant decrease in LC3BII levels was demonstrated when compared to NM-*LRRK2*^*G2019S*^ subjects at 4 and 8 h of bafilomycin treatment in glucose media (− 79 to 86%, *p *= *0.042* and − 71.26%, *p *= *0.22* respectively) (Fig. 4d).

## Discussion

The main contribution of this study was the exhaustive analysis of mitochondrial phenotype and autophagic print of fibroblasts from *LRRK2*^*G2019S*^ mutation carriers with and without clinical evidence of parkinsonism in glucose and galactose media. Briefly, this study suggests that the exhaustion of mitochondrial bioenergetic and autophagic reserve play a role in the onset of *LRRK2*^*G2019S*^-associated PD.

Mainly, in standard conditions (glucose), the NM-*LRRK2*^*G2019S*^ fibroblasts showed a preserved mitochondrial phenotype, except for a significant disruption of MMP that may represent an early event in PD [14, 38–40]. In response to mitochondrial-challenging conditions (galactose), a pattern of mitochondrial enzymatic and respiratory function enhancement was observed, resulting in an increase of ATP production and morphological features characteristic of improved mitochondrial dynamics. In PD-*LRRK2*^*G2019S*^ fibroblasts, bias toward deranged mitochondrial phenotype were found in glucose media, such as decreased CI enzyme function, oxygen consumption and ATP production, and increased oxidative stress. Exposure to galactose revealed an inefficient increase in mitochondrial function, trends to increase oxidative stress and deranged mitochondrial dynamics. The most differential response between NM-*LRRK2*^*G2019S*^ subjects and PD-*LRRK2*^*G2019S*^ patients in mitochondrial functional and dynamic parameters was shown in response to mitochondrial-challenging conditions. In accordance to literature, mitochondrial defects were evidenced in galactose media [22], which leads us to hypothesize that, given their oxidative metabolism and postmitotic nature, neurons may be permanently damaged by mitochondrial and autophagic alterations that in peripheral tissues may be negligible [41, 42]. Ergo, a deficient mitochondrial functional reserve may underlie the onset of *LRRK2*^*G2019S*^-associated PD. The link between mitochondrial health and autophagy initiation may be deduced from these findings, but needs to be assessed in more detail.

Recent reports have described differential mitochondrial functional phenotypes in idiopathic and some monogenic forms of PD [43–45]. Our study partially reproduces the mitochondrial phenotype described by Mortiboys et al. [17], where CI enzyme activity, oxygen consumption and ATP levels progressively decreased in NM-*LRRK2*^*G2019S*^ and PD-*LRRK2*^*G2019S*^. Papkovskaia et al. [39] also found decreased ATP levels and depolarization in fibroblasts of PD-*LRRK2*^*G2019S*^.

Oxidative stress is a hallmark of mitochondrial involvement on PD and neurodegeneration [11, 46]. Our results are in accordance with Grünewald et al. [40], who found increased levels of the antioxidant superoxide dismutase, but no significant alteration of oxidative stress in fibroblasts of NM-*LRRK2*^*G2019S*^, suggesting the existence of compensatory mechanisms against early-stage production of ROS. Liou et al. [47], reported a protective effect against oxidative stress-mediated apoptosis mediated by wild type LRRK2 which is lost in PD-associated mutations. In our study, trends towards reduced apoptosis in NM-*LRRK2*^*G2010S*^ in spite of increased oxidative stress was found, which suggests the existence of other protecting mechanisms that are yet to be described.

This study corroborated the impairment in mitochondrial dynamics in PD-*LRRK2*^*G2019S*^ patients described by others [17, 31, 40, 48]. Additionally, a healthier pattern in mitochondrial dynamics in NM-*LRRK2*^*G2019S*^ subjects was demonstrated. Whether alterations in mitochondrial dynamics are the cause or consequence of mitochondrial dysfunction, and the mechanism by which LRRK2 mediates such alterations, is still a matter of debate [3].

Trends towards upregulation in autophagy in NM-*LRRK2*^*G2019S*^ were observed, which may be explained by any of the previously described effects of *LRRK2*^*G2019S*^ mutation in autophagy [49–51] or by an increased attempt to eliminate damaged sub cellular compounds. A further increase in autophagy initiation signalling in PD-*LRRK2*^*G2019S*^ patients was found, probably in response to mitochondrial dysfunction and the resulting decrease in ATP levels evidenced by galactose exposure [13, 52].

However, the inability to continue with autophagosome formation and closure, and the resulting accumulation of damaged mitochondria among other waste product of cell metabolism, may explain the pathologic state in this group. Further studies should confirm the present findings, where mitophagy and autophagic flux may be characterized in more detail [53].

This is the first study reporting that the exposition of fibroblasts from NM-*LRRK2*^*G2019S*^ to mitochondrial challenging conditions tends to enhance mitochondrial performance, even above the values of controls. We hypothesize that such improvement represents a biological rescue mechanism characteristic of NM-*LRRK2*^*G2019S*^, which may protect from the development of symptoms by compensating an increased bioenergetic cell demand or early mitochondrial damage. It may be deduced that mitochondrial and autophagic optimal function could have a protective role in mutation carriers who have not yet developed symptoms of PD. Mitochondria from NM-*LRRK2*^*G2019S*^ may retain the capacity to raise their activity until a maximal threshold beyond which PD clinical manifestations can appear.

On account of the above, our results suggest that *LRRK2*^*G2019S*^-mutation by itself may not determine the development of clinical manifest PD [2, 52, 54], in accordance with other previous studies where NM-*LRRK2*^*G2019S*^ subjects perform as good as controls in motor [18], neuropsychological [55] and neuroimaging tests [56]. Scientific efforts focused on promoting mitochondrial optimal performance and autophagy regulation should be translated into prophylactic measures which modify the natural history of PD.

In regard to the experimental model used in this study, the exposure to oxidative metabolism through galactose media unveiled a differential mitochondrial phenotype that may reproduce that of neurons. Herein, we suggest that alternative models of PD should also be tested in oxidative conditions [41].

Some limitations in the present study must be acknowledged. Although the study of rare forms of PD due to inherited mutations reduces the potential study of bigger cohorts, the small sample size of this study may explain a common pitfall of the reported outcomes, which is the limited number of significant differences. Additionally, the inter-individual variability in environmental conditions potentially influencing molecular parameters may also hinder the homogeneity of the distinct groups. There is a positive correlation between age and PD onset in *LRRK2*^*G2019S*^ mutation carriers [2], suggesting that some NM-*LRRK2*^*G2019S*^ may eventually develop PD. However, it must be emphasized that no age related differences in mitochondrial and autophagic parameters were observed. Finally, many other molecular triggers may be taking place in PD, where the loss of dopaminergic neurons may be the common manifestation of a complex disease where mitochondrial alterations and autophagic deregulation are only one of manifold molecular events leading to neurodegeneration.

## Conclusions

Enhanced mitochondrial performance of NM-*LRRK2*^*G2019S*^ in mitochondrial-challenging conditions and upregulation of autophagy suggests that an exhaustion of mitochondrial bioenergetic and autophagic reserve, may contribute to the development of PD in *LRRK2*^*G2019S*^ mutation carriers.

## Additional files

**Additional file 1: Materials and methods.** This part of additional material is provided in a separate word document named “Additional material, materials and methods”.

**Additional file 2: Table S1.** Clinical characteristics of PD-*LRRK2*^*G2019S*^ patients (n=7). The following legend accompanies the table: NM-*LRRK2*^*G2019S*^: Non-manifesting carriers of *LRRK2*^*G2019S*^-mutation; PD-*LRRK2*^*G2019S*^: patients with *LRRK2*^*G2019S*^-mutation and clinically manifest PD.

**Additional file 3: Figure S1.** Principal component analysis (PCA) for patients and controls in the different media. Variables factor map showing that individual variability in glucose media **(A)** was best expressed by mitochondrial dynamics parameters -circularity and mitochondrial network- for component 1 (horizontal axis), and autophagy parameters for component 2 (vertical axis). When subjected to mitochondrial challenging conditions **(B)**, circularity expressed the greatest variability for component 1 (horizontal axis), and MMP better expressed component 2 variability (vertical axis). The longer vectors and those which are more aligned to the corresponding axis (depicted as dotted lines) are the ones with the greatest variability among individuals, which interestingly have been documented to be associated with PD, despite none of the parameters represent a greater variability between groups. MtDNA: mitochondrial-DNA; mtRNA: mitochondrial-RNAVDAC/βactin: Mitochondrial content; COXII/βactin: Mitochondrial encoded protein content COXIV/βactin; Nuclear encoded protein content; CI: Complex I enzymatic function; PMox: oxygen consumption stimulated by pyruvate-malate; CIV: Complex IV enzymatic function; MMP: Mitochondrial membrane potential; OE: Oxidative stress; Circ: Circularity; P62: Autophagy substrate LC3BI: Autophagy receptor, basal form; LC3BII: Lipidated form of the autophagy receptor, LC3BII/LC3BI: autophagic turnover, LC3BII/P62: autophagic flux.

**Additional file 4: Table S2.** Raw data of mitochondrial phenotype and autophagic print parameters. The following legend accompanies the table: Glu: glucose; Gal: galactose; NM-*LRRK2*^*G2019S*^: Non-manifesting carriers of *LRRK2*^*G2019S*^-mutation; PD- *LRRK2*^*G2019S*^: patients with *LRRK2*^*G2019S*^ -mutation and clinically manifest PD; mtDNA: Mitochondrial DNA; mtRNA: Mitochondrial RNA; CI: Complex I; O2 consumption stimulated for CI: Oxygen consumption stimulated for Complex I substrates (pyruvate, malate and glutamate); CIV: Complex IV. Pa. P value when comparing NM-*LRRK2*^*G2019S*^ vs. controls; Pb. P value when comparing PD- *LRRK2*^*G2019S*^ vs. controls; Pc. P value when comparing NM-*LRRK2*^*G2019S*^ vs. PD-*LRRK2*^*G2019S*^.

**Additional file 5: Figure S2.** Genetics and mitochondrial protein synthesis. Results are represented by means ± SEM, comparing controls (white bars), non-manifesting carriers of *LRRK2*^*G2019S*^-mutation, (NM-*LRRK2*^*G2019S*^, grey bars) and patients with *LRRK2*^*G2019S*^-mutation and clinically manifest PD (PD-*LRRK2*^*G2019S*^, black bars) in glucose and galactose media. **A.** Mitochondrial DNA was conserved in both groups and media. **B.** Mitochondrial RNA levels were significantly decreased in NM-*LRRK2*^*G2019S*^ when compared to controls in galactose media **C-E**. Cell growth, mitochondrial content and protein synthesis did not show differences between groups, in either condition. **F.** Representative image of Western Blot of mitochondrial proteins in either glucose (Glu) or galactose (Gal) media of the three cohorts studied, the original Blott has been cropped for its better visualization, complete images can be provided at request.

## Abbreviations

PD: Parkinson’s disease
LRRK2: leucine rich repeat kinase 2
*LRRK2*^*G2019S*^: *LRRK2*^*G2019S*^-mutation
NM-*LRRK2*^*G2019S*^: *LRRK2*^*G2019S*^-carriers without PD symptoms
PD-L*RRK2*^*G2019S*^: *LRRK2*^*G2019S*^-carriers diagnosed with PD
ATP: adenosine triphosphate
MMP: mitochondrial membrane potential
mtDNA: mitochondrial DNA
mtRNA: mitochondrial RNA
VDAC: voltage-dependent anion channel
COXII: cytochrome oxidase subunit II
COXIV: cytochrome oxidase subunit IV
MRC: mitochondrial respiratory chain
CI: Complex I of the MRC
CIV: Complex IV of the MRC
CS: citrate synthase
PMox: oxygen consumption was assessed through pyruvate-malate oxidation
MDA: malondialdehyde
HAE: 4-hydroxyalkenal
ROS: reactive oxygen species
Circ: circularity
AR: aspect ratio
FF: form factor
(SQSTM/p62)/β-actin: autophagy substrate
LC3BII/β-actin: autophagy receptor, lipidated form
SEM: standard error of the mean
PCA: principal component analysis
