# Supplementary material for: Exhaustion of mitochondrial and autophagic reserve may contribute to the development of LRRK2G2019S-Parkinson’s disease
Source: J Transl Med. 2018 Jun 8;16:160. doi: 10.1186/s12967-018-1526-3 (PMC5994110; doi:10.1186/s12967-018-1526-3)
Supplement: Supplementary file 1 — Additional file 1: Materials and methods. This part of additional material is provided in a separate word document named “Additional material, materials and methods”. [file 12967_2018_1526_MOESM1_ESM.docx]

**ADDITIONAL MATERIAL**

**MATERIALS AND METHODS**

1. Specific composition of glucose media was: Dulbecco’s modified Eagle’s medium (DMEM) from Gibco™, containing 25mM glucose, 4mM L-glutamine, 10% FBS and 1% penicillin-streptomycin. Specific composition of galactose media was: DMEM without glucose or phenol-red from Gibco™, supplemented with 10mM D-galactose, 2mM glutamine, 4% FBS, 1mM sodium pyruvate and 1% penicillin-streptomycin ^[23]^. All functional assays were performed with cells younger than passage 10. Experiments were run in parallel including one subject from each cohort, at the same passage, both in glucose and galactose media.
2. *Mitochondrial DNA content and RNA levels:* After extraction, mtDNA was analyzed in a 96-well plate by the amplification of a fragment from the conserved 12SrRNA mitochondrial gene with respect to the nuclear constitutive gene RNAseP, using real-time quantitative PCR (RTqPCR) from Applied Biosystems™ (Foster City, California, USA). Results were expressed in relative units as the ratio between mtDNA to nuclear DNA (mt12SrRNA/nRNaseP). Total RNA was extracted by affinity micro columns using SpeedTools Total RNA Extraction Kit® (Biotools™, B&M labs, Madrid, Spain), following manufacturer’s instructions. After quantification, reverse transcription was performed by using random hexamer primers before the RTqPCR experiment. Mitochondrial RNA content was afterwards analysed through the amplification of ND2/18SrRNA cDNA. The results were expressed as the ratio between mitochondrial to nuclear RNA (mt12SrRNA/nRNaseP) ^[24]^.
3. *Mitochondrial content and respiratory chain enzymatic activities*: Citrate synthase (CS) was measured by spectrophotometry at 37ºC and 412nm, following Spanish standardized national procedures [31]. Results were expressed as nanomoles of product generated per minute and milligram of protein (nmols/min*mg protein). CI and CIV enzymatic activities of the MRC were measured by spectrophotometry 340 nm and 550 nm to respectively, following Spanish standardized national procedures [31]. Measurement of CI activity required previous treatment of cells with triton and digitonin detergents to obtain specific mitochondrial NADH-dehydrogenase activity. Enzymatic activities were calculated as nanomoles of consumed substrate per minute and milligram of protein (nmols/min*mg protein) and then normalized to CS activity to relativize the enzymatic activity by mitochondrial content.
4. *Oxygen consumption through pyruvate-malate-glutamate oxidation (PMox):* Results were expressed as nanomoles of consumed oxygen per minute and milligram of protein (nmols/min*mg protein).
5. *Total cellular ATP:* Total cellular ATP content was measured by luminescence using Luminescent ATP detection assay kit® #ab113849; Abcam™ (Cambridge, United Kingdom). Results were normalized by protein levels and expressed as picomols of ATP per microliter and milligram of cell protein (pmol ATP /μl*mg prot).
6. *Mitochondrial Membrane Potential:* MMP was evaluated by using 5,5′,6,6′-tetrachloro-1,1′,3,3′ tetraethyl benzimidazolocarbocyanine iodide dye (JC1), by quantifying the percentage of fibroblasts with depolarized mitochondria from the total number, as previously described [33]. The results were expressed as the percentage of cells with depolarized mitochondria, with respect to total cells (% cells with depolarized mt/total cells).
7. *Oxidative damage:* Lipid peroxidation was measured as an indicator of oxidative damage of reactive oxygen species (ROS) into cellular lipid compounds using the Calorimetric assay for Lipid Peroxidation Kit #21012; Bioxytech® LPO-586™, *Oxis*Research™ (Portland, OR, USA), by the spectrophotometric measurement of malondialdehyde (MDA) and 4-hydroxyalkenal (HAE), both products derived from fatty acid peroxide decomposition, at 586 nm [33]. Results were normalized by protein content, and expressed as MDA and HAE concentration (in micromoles per litre) per milligram of cell protein (µM MDA + 4-HAE / mg prot).
8. *Apoptotic rate:* After double staining for annexin V and propidium iodide apoptotic rate was quantified by flow cytometry, as previously described [35]. Results were reported as the percentage of double stained cells, with respect to total cells (% stained cells/total cells)
9. *Mitochondrial dynamics*: Cells were seeded in a 16-well glass slide (Nunc™ #178599 Lab-Tek® Chamber Slide™, Austin, USA) in both, glucose and galactose media, for 24 h. Mitochondrial network was stained by incubation with rabbit Tom20 Antibody (FL-145) (Santa Cruz Biotechnology, Dallas, USA) and secondary antibody donkey anti-rabbit Alexa Fluor® 488 IgG (Life Technologies Europe, NL). Wheat Germ Aglutinin Alexa Fluor® 594 conjugate (Life Technologies Europe, NL) and TO-PRO 3® iodide #T3605 (Life Technologies Europe, NL) were used for cytoplasm immunostaining and nuclei detention, respectively. Images were obtained with a Leica™ TCS SP5 laser scanning confocal system using 63X oil immersion objective. Analysis was performed using 2.5X zoom for each cell with Image J [38] software. The following parameters were assessed for mitochondrial dynamics evaluation: (i) Mitochondrial network or mitochondrial content: Total number of mitochondria/total cell area [39]; higher mitochondrial network values are considered sign of healthy mitochondria. (ii) Circularity (Circ) or mitochondrial isolation: 4π.area/perimeter^2^; circular mitochondria have less interaction sites with other mitochondria, thus, Circ=1 refers to poor mitochondrial dynamics of isolated mitochondria. (iii) Aspect Ratio (AR) or mitochondrial elongation: major/minor axis, AR=1 indicates a perfect circle, AR increases as mitochondria elongate and become more elliptical, which is considered a beneficious sign of mitochondrial dynamics. (iv) Form factor (FF) or mitochondrial branching: Circ-1; FF=1 corresponds to a circular, unbranched mitochondrion and high FF values indicate a branched, connected and active mitochondrion.
10. *Reagents and antibodies used for Western Blot*
    1. Anti-VDAC1/Porin antibody (37KDa; 1:1000; Abcam, ab14734 Cambridge, UK)
    2. MTCO2 antibody (25.6 KDa;1:1000; Invitrogen™ #A-6404; Carlsbad, CA, USA)
    3. Complex IV Monoclonal Antibody (15KDa; 1:1000; Invitrogen™ #A-6404; Carlsbad, CA, USA)
    4. β-actin Antibody (47 KDa; 1:30.000; Sigma-Aldrich®, #A2228, St.Louis, MO, USA)
    5. Halt Protease Inhibitor Single-Use Cocktail EDTA-Free, #78425, ThermoScientific™ (Rockford, IL, USA).
    6. Bio-Rad’s TGX™ Fast Cast™ Acrylamide Kit, 7.5/12%, #1610181 polyacrylamide gels (Biorad, USA)
    7. Nitrocellulose membranes: iBlot® Gel Transfer Stacks, #IB301001, Invitrogen™, Carlsbad, CA, USA)
    8. Anti-SQSTM1/p62 antibody (62 KDa; 1:4000; Abcam, ab56416, Cambridge, UK)
    9. LC3B Antibody (14 and 16 KDa; 1:250, Cell Signaling® #2775, Denver, MA, USA)
11. *Imunocytochemistry for autophagy characterization:* Cells were seeded in a 16-well glass slide (Nunc™ #178599 Lab-Tek® Chamber Slide™, Austin, USA) in both, glucose and galactose media, for 24 h. Degradation of autophagolysosomes was blocked by adding 100nM Bafilomycin A1 from *Streptomyces griseus* (Sigma-Aldrich® #B1793 SIGMA, Missouri, USA) for 6 hours. Autophagosomes were stained by incubation with Anti-LC3 pAB (MBL International® #PM036, Massachusets, USA) and secondary antibody donkey anti-rabbit Alexa Fluor® 488 IgG (Life Technologies Europe, NL). Counterstain with DAPI was performed for nucleus staining (DAPI Fluoromount-G® #0100-20, Southern Biotech, Alabama, USA). Images were obtained with a Zeis LSM 880 laser scanning confocal system using 63X oil immersion objective.
12. *Protein content:* The bicinchoninic acid (BCA) assay was used to calculate the total protein cell content: (Pierce BCA Protein Assay Kit #23225; Thermo Scientific™, Rockford, IL, USA).
