# Supplementary material for: Exhaustion of mitochondrial and autophagic reserve may contribute to the development of LRRK2G2019S-Parkinson’s disease
Source: J Transl Med. 2018 Jun 8;16:160. doi: 10.1186/s12967-018-1526-3 (PMC5994110; doi:10.1186/s12967-018-1526-3)
Supplement: Supplementary file 2 — Additional file 2: Table S1. Clinical characteristics of PD-LRRK2G2019S patients (n=7). The following legend accompanies the table: NM-LRRK2G2019S: Non-manifesting carriers of LRRK2G2019S-mutation; PD-LRRK2G2019S: patients with LRRK2G2019S-mutation and clinically manifest PD. [file 12967_2018_1526_MOESM2_ESM.docx]

**Additional Table S1.** Clinical characteristics of PD-*LRRK2^G2019S^* patients (n=7)

NM-*LRRK2^G2019S^*: Non-manifesting carriers of *LRRK2^G2019S^*-mutation; PD-*LRRK2^G2019S^*: patients with *LRRK2^G2019S^*-mutation and clinically manifest PD.

|  | | **PRESENT** | **NOT PRESENT** | **UNKNOWN** |
| --- | --- | --- | --- | --- |
| MUTATION | *G2019S* | 7 | 0 | 0 |
| FAMILY HISTORY | Parkinsonism | 5 | 0 | 2 |
|  | Dementia | 0 | 5 | 2 |
| DIAGNOSTIC CRITERIA | Unilateral tremor at beginning | 6 | 0 | 1 |
|  | Tremor at rest | 5 | 1 | 1 |
|  | Progressive disease | 6 | 0 | 1 |
|  | Asymmetric persistence | 5 | 1 | 1 |
| CLINICAL FEATURES AT TIME OF BIOPSY | Tremor at rest | 5 | 1 | 1 |
|  | Dementia | 1 | 5 | 1 |
|  | Deep brain stimulation | 2 | 3 | 2 |
|  | Clinical course >10 years | 4 | 1 | 2 |
| RESPONSE TO TREATMENT | First response to Ldopa | 5 | 0 | 2 |
|  | Response to Ldopa>5 years | 3 | 0 | 4 |
