# Supplementary figures and images for: Exhaustion of mitochondrial and autophagic reserve may contribute to the development of LRRK2G2019S-Parkinson’s disease
Source: J Transl Med. 2018 Jun 8;16:160. doi: 10.1186/s12967-018-1526-3 (PMC5994110; doi:10.1186/s12967-018-1526-3)

## A. GLUCOSE

Variables factor map (PCA)

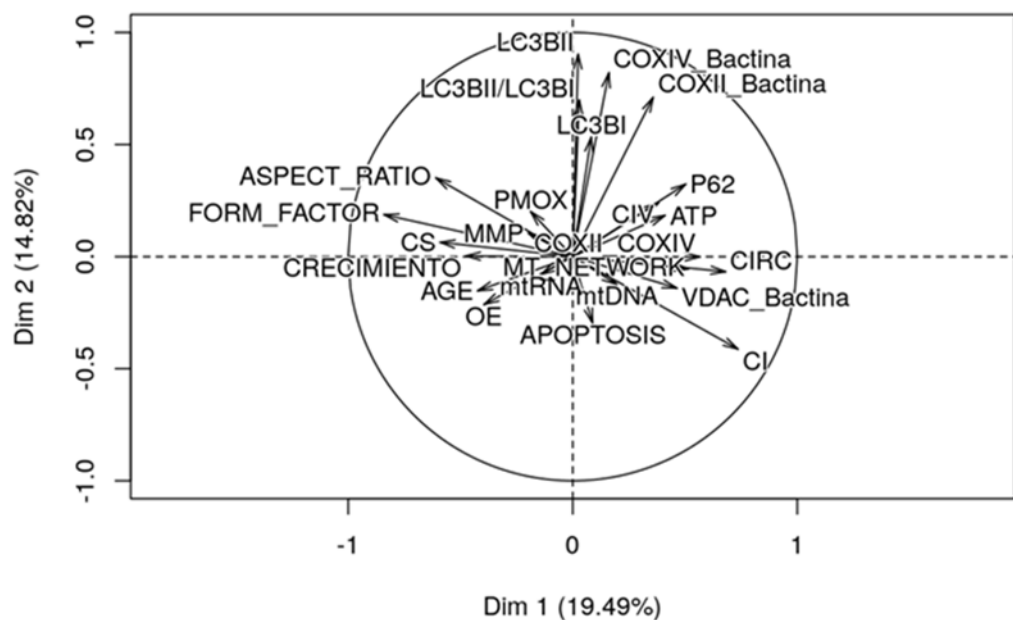

## B. GALACTOSE

Variables factor map (PCA)

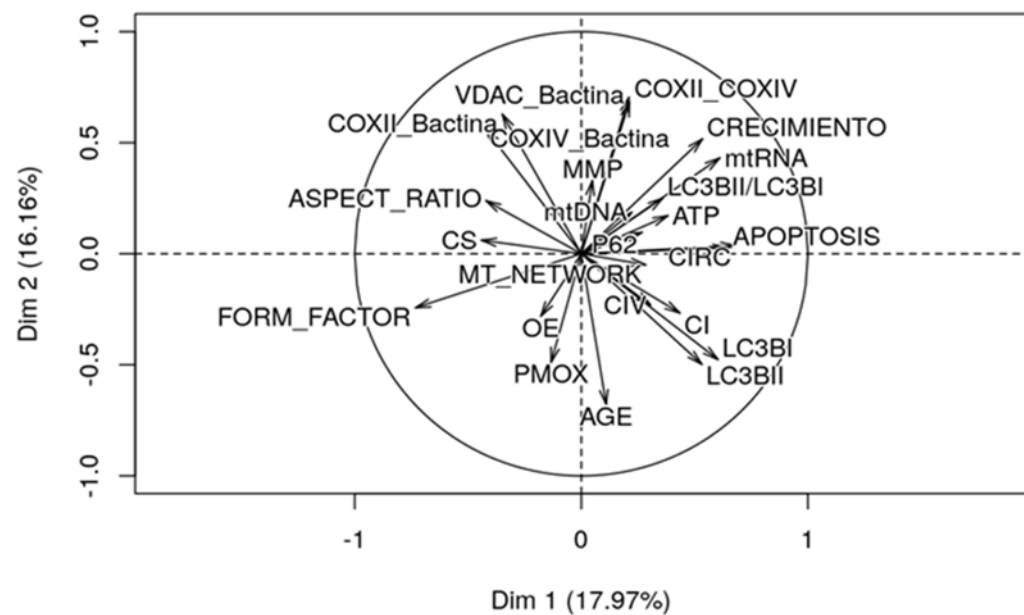

Supplement: Supplementary file 3 — Additional file 3: Figure S1. Principal component analysis (PCA) for patients and controls in the different media. Variables factor map showing that individual variability in glucose media (A) was best expressed by mitochondrial dynamics parameters -circularity and mitochondrial network- for component 1 (horizontal axis), and autophagy parameters for component 2 (vertical axis). When subjected to mitochondrial challenging conditions (B), circularity expressed the greatest variability for component 1 (horizontal axis), and MMP better expressed component 2 variability (vertical axis). The longer vectors and those which are more aligned to the corresponding axis (depicted as dotted lines) are the ones with the greatest variability among individuals, which interestingly have been documented to be associated with PD, despite none of the parameters represent a greater variability between groups. MtDNA: mitochondrial-DNA; mtRNA: mitochondrial-RNAVDAC/βactin: Mitochondrial content; COXII/βactin: Mitochondrial encoded protein content COXIV/βactin; Nuclear encoded protein content; CI: Complex I enzymatic function; PMox: oxygen consumption stimulated by pyruvate-malate; CIV: Complex IV enzymatic function; MMP: Mitochondrial membrane potential; OE: Oxidative stress; Circ: Circularity; P62: Autophagy substrate LC3BI: Autophagy receptor, basal form; LC3BII: Lipidated form of the autophagy receptor, LC3BII/LC3BI: autophagic turnover, LC3BII/P62: autophagic flux. [file 12967_2018_1526_MOESM3_ESM.pdf]
