# Supplementary material for: Exhaustion of mitochondrial and autophagic reserve may contribute to the development of LRRK2G2019S-Parkinson’s disease
Source: J Transl Med. 2018 Jun 8;16:160. doi: 10.1186/s12967-018-1526-3 (PMC5994110; doi:10.1186/s12967-018-1526-3)
Supplement: Supplementary file 5 — Additional file 5: Figure S2. Genetics and mitochondrial protein synthesis. Results are represented by means ± SEM, comparing controls (white bars), non-manifesting carriers of LRRK2G2019S-mutation, (NM-LRRK2G2019S, grey bars) and patients with LRRK2G2019S-mutation and clinically manifest PD (PD-LRRK2G2019S, black bars) in glucose and galactose media. A. Mitochondrial DNA was conserved in both groups and media. B. Mitochondrial RNA levels were significantly decreased in NM-LRRK2G2019S when compared to controls in galactose media C-E. Cell growth, mitochondrial content and protein synthesis did not show differences between groups, in either condition. F. Representative image of Western Blot of mitochondrial proteins in either glucose (Glu) or galactose (Gal) media of the three cohorts studied, the original Blott has been cropped for its better visualization, complete images can be provided at request. [file 12967_2018_1526_MOESM5_ESM.pdf]

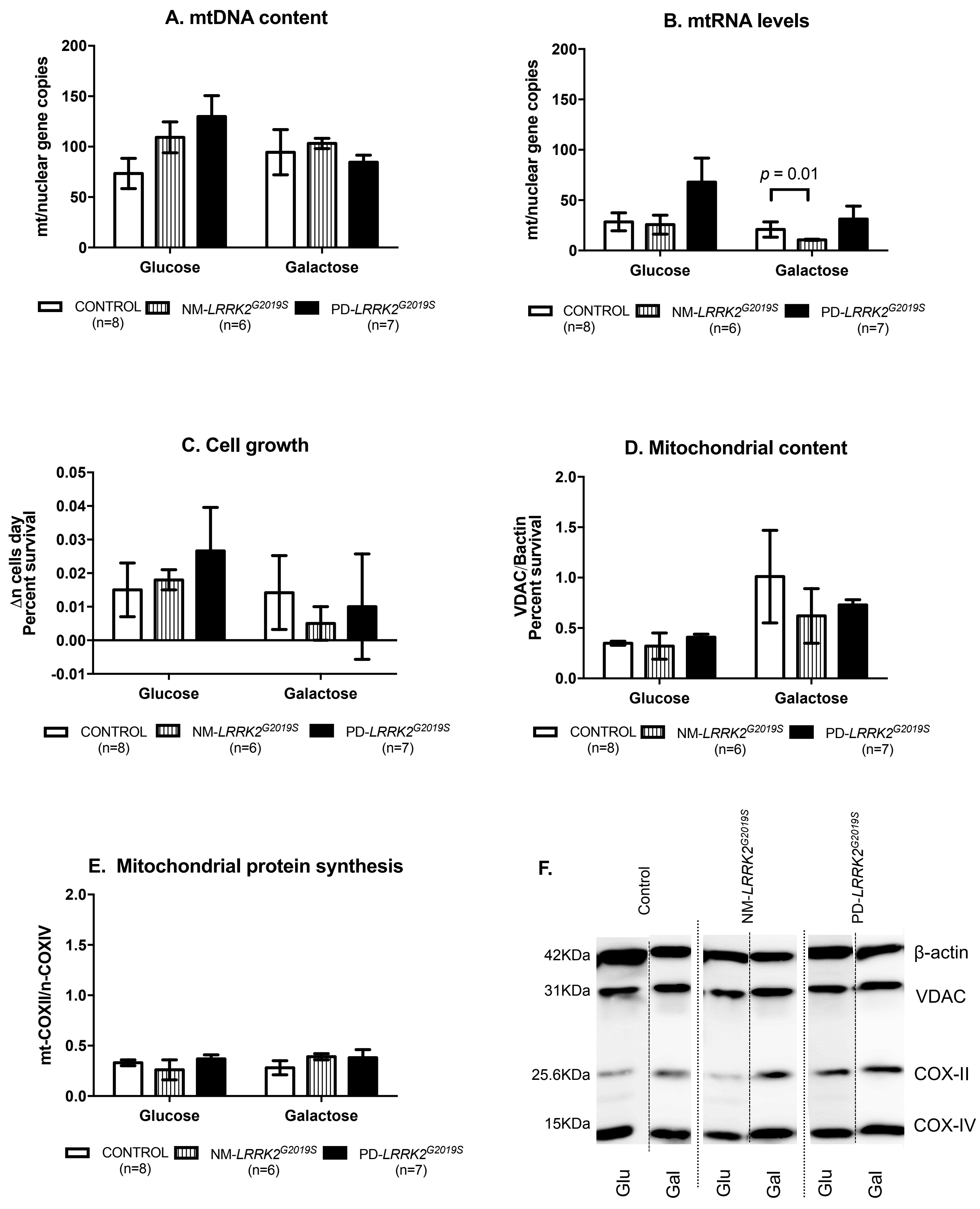
A. mtDNA content
mt/nuclear gene copies
200
150
100
50
0
Glucose
Galactose
CONTROL (n=8)
NM-LRRK2G2019S (n=6)
PD-LRRK2G2019S (n=7)
B. mtRNA levels
mt/nuclear gene copies
200
150
100
50
0
p = 0.01
Glucose
Galactose
CONTROL (n=8)
NM-LRRK2G2019S (n=6)
PD-LRRK2G2019S (n=7)
C. Cell growth
Δn cells day
Percent survival
0.05
0.04
0.03
0.02
0.01
0.00
-0.01
Glucose
Galactose
CONTROL (n=8)
NM-LRRK2G2019S (n=6)
PD-LRRK2G2019S (n=7)
D. Mitochondrial content
VDAC/Bactin
Percent survival
2.0
1.5
1.0
0.5
0.0
Glucose
Galactose
CONTROL (n=8)
NM-LRRK2G2019S (n=6)
PD-LRRK2G2019S (n=7)
E. Mitochondrial protein synthesis
mt-COXII/n-COXIV
2.0
1.5
1.0
0.5
0.0
Glucose
Galactose
CONTROL (n=8)
NM-LRRK2G2019S (n=6)
PD-LRRK2G2019S (n=7)
F.
Control
NM-LRRK2G2019S
PD-LRRK2G2019S
42KDa
31KDa
25.6KDa
15KDa
β-actin
VDAC
COX-II
COX-IV
Glu
Gal
Glu
Gal
Glu
Gal
